# Enhancing Toughness of PLA/ZrP Nanocomposite through Reactive Melt-Mixing by Ethylene-Methyl Acrylate-Glycidyl Methacrylate Copolymer

**DOI:** 10.3390/polym14183748

**Published:** 2022-09-07

**Authors:** Chuanbiao Zhu, Xiang Lu, Yi Li, Yanhong Deng, Jiuling Lu, Zhigang Liu, Hao Wu, Yi Tong, Jinping Qu

**Affiliations:** 1 Key Laboratory of Material Chemistry for Energy Conversion and Storage of Ministry of Education, School of Chemistry and Chemical Engineering, Huazhong University of Science & Technology, Wuhan 430074, China; 2 Hubei Engineering Research Center for Biomaterials and Medical Protective Materials, Huazhong University of Science & Technology, Wuhan 430074, China; 3 Hubei Key Laboratory of Material Chemistry and Service Failure, School of Chemistry and Chemical Engineering, Huazhong University of Science & Technology, Wuhan 430074, China; 4 COFCO (Jilin) Bio-Chemical Technology Co., Ltd., Changchun 130033, China; 5 Suzhou COFCO Biochemical Co., Ltd., Suzhou 234000, China; 6 Key Laboratory of Polymer Processing Engineering (South China University of Technology), Ministry of Education, Guangzhou 510641, China

**Keywords:** poly(lactic acid), zirconium phosphate, super-tough, nanocomposite, compatibility

## Abstract

The nanofiller zirconium phosphate (ZrP) was mixed into poly(lactic acid) (PLA) to ameliorate its thermal stability. The elastomer ethylene-methyl acrylate-glycidyl methacrylate copolymer (E-MA-GMA) was introduced into the PLA/ZrP nanocomposite through melt-mixing to improve its toughness and obtain a super-tough PLA/ZrP/E-MA-GMA nanocomposite. The impact strength of the PLA/ZrP/E-MA-GMA nanocomposite, with a composition ratio of 72/3/25, was improved to 71.5 kJ/m^2^, about 25 times greater than the impact strength of pure PLA. The dynamic mechanical analysis (DMA) confirmed that E-MA-GMA has excellent compatibility with the matrix of PLA. A typical core–shell structure that can cause massive shear-yielding deformation was characterized by transmission electron microscopy (TEM), which gave the nanocomposite excellent toughness.

## 1. Introduction

With the increasing severity of environmental pollution, petroleum-based polymer materials are gradually being replaced by biodegradable polymer materials, such as films, fibers, packaging, containers, and biomedical materials [1,2,3,4]. As a fully biodegradable material [5], PLA has attracted increasing interest due to its merits of fine biocompatibility, excellent transparency, renewability, complete degradation, and high mechanical strength [6,7,8]. Although PLA possesses the above excellent properties, the brittleness of PLA seriously restricts its further application [9]. The fine toughness of PLA is indispensable for developing PLA composites with the desired performances [10]. Therefore, PLA-based composites with super toughness have attracted a substantial amount of research interest over the past few years. Particularly, “super-toughness” performance is highly pursued for PLA application areas, mainly for applications in packaging and automotive interior parts [11]. Consequently, researchers have put forward numerous improvement measures, such as plasticization [12], physical mixing [13,14,15,16,17,18], and copolymerization [19,20,21], to ameliorate the toughness of PLA. However, the above methods cannot simultaneously overcome the related problems of a complex reaction and low interfacial compatibility; most of the polymers showed poor miscibility with PLA, which led to an unsatisfactory toughening effect. In recent years, the reactive mixing of the elastomer ethylene-methyl acrylate-glycidyl methacrylate copolymer (E-MA-GMA) with PLA through melt-mixing has been demonstrated to be an extraordinarily effective method for ameliorating the toughness of PLA. Zhang et al. [10] explored the formulation of super tough PLA composites via the melt blending of PLA/E-MA-GMA/PEBA (poly(ether-b-amide)), and reported that the PLA/EMA-GMA binary blends displayed good interface compatibility. Wu et al. [22] also indicated that the strong interfacial adhesion provided by E-MA-GMA was responsible for super tough PLA-based composites. They found that the carboxyl and hydroxyl groups at the ends of the PLA molecular chains will react with E-MA-GMA through epoxy groups on its molecular chains during the mixing process; consequently, the toughness of the PLA matrix was significantly improved [23,24,25].

In addition to brittleness, another urgent challenge that must be solved with respect to PLA is its poor thermal stability. PLA has been severely limited in the processing and application fields due to its low thermal stability. Therefore, PLA must possess an adequate thermal stability for preventing the degradation of its Mw and physical properties. In recent years, researchers found that inorganic fillers can be added into polymer materials to prepare nanocomposites so as to overcome these issues. Under the premise of maintaining a light weight, the addition of a small amount of inorganic fillers is a simple and valid method used to ameliorate polymers’ properties, such as thermal stability, optical transparency, flame retardancy, the gas barrier, and the fine mechanical characteristics, in order to broaden their application [26,27]. Among these inorganic fillers, zirconium phosphate (ZrP) is a kind of lamellar material with a large specific surface area, a perfect particle size, and good chemical and thermal stability [28,29,30]. Benefiting from these properties of ZrP, the thermal stability of the PLA-based nanocomposites can be effectively ameliorated. Unfortunately, as with most inorganic fillers, the introduction of ZrP alone cannot solve the problem of the poor flexibility of PLA. Moreover, the incompatibility between ZrP and PLA is also a great challenge to the properties of PLA-based nanocomposites. Based on the above considerations, we designed an experiment to introduce E-MA-GMA into PLA/ZrP nanocomposites to improve their defection so that the epoxy group of elastomer E-MA-GMA could react with −OH group of ZrP nanofiller. Furthermore, the epoxy group of the elastomer E-MA-GMA could also react with the end groups of −COOH and −OH on PLA’s molecular chains. The reactions among PLA, E-MA-GMA, and ZrP improve the interface combination between ZrP and PLA, which grants the nanocomposite excellent properties.

In this work, we prepared super-tough PLA/ZrP/E-MA-GMA nanocomposites with a good thermal stability through reactive melt-mixing. The thermal stability of the PLA/ZrP/E-MA-GMA nanocomposites was characterized by a thermogravimetric analysis (TGA) and differential scanning calorimetry (DSC). The reaction mechanisms among PLA, E-MA-GMA, and ZrP were revealed by Fourier-transform infrared spectroscopy (FTIR). Transmission electron microscope (TEM) and scanning electron microscope (SEM) were used to analyze the micro morphology of the PLA/ZrP/E-MA-GMA nanocomposites, which revealed the toughening mechanism of the E-MA-GMA on PLA/ZrP composites. The mechanical properties of the PLA/ZrP/E-MA-GMA nanocomposite were shown via tensile and impact strength testing.

## 2. Materials and Methods

### 2.1. Materials

The PLA, labeled as 4032D, was purchased from Nature Works Co. Ltd. (Minnetonka, MN, USA); its density was 1.24 g/cm^3^, and its molecular weight and polydispersity index were 1.7 × 10^5^ g/mol and 1.74, respectively. The E-MA-GMA, labeled as Lotader AX 8900, with 8% glycidyl methacrylate, 24% acrylic ester, and 68% ethylene, was manufactured by Arkema Company. The lamellar inorganic nanofiller ZrP, with more than 40% content of ZrO_2_, was supplied by Mianzhu Yaolong Chemical Co. Ltd. (Deyang. China). The average data of lamellar thickness and particle size for the ZrP nanofillers were 83.5 nm and 1.7 μm, and the density of the nanofiller was 1.6 g/cm^3^.

### 2.2. Sample Preparation

An oven was used to dry the pellets of PLA and the powder of ZrP at 75 °C for 10 h. Afterwards, the mixture of PLA/ZrP (3 wt%) with different contents (5 wt%, 15 wt%, 25 wt%) of E-MA-GMA were melt-mixed in a Brabender W50E mixer. The speed of the mixer was 60 r/min, while the processing temperature was 200 °C and mixing time was 6 min. Then, the compounds were compressed into sheets under the pressure of 10 Mpa and temperature of 200 °C by a QBL-350 compression-molding machine, which was produced by China Wuxi No. one Rubber and Plastic Machinery Co., Ltd. (Taizhou, China).

### 2.3. Reaction Mechanism

**Figure 1:**
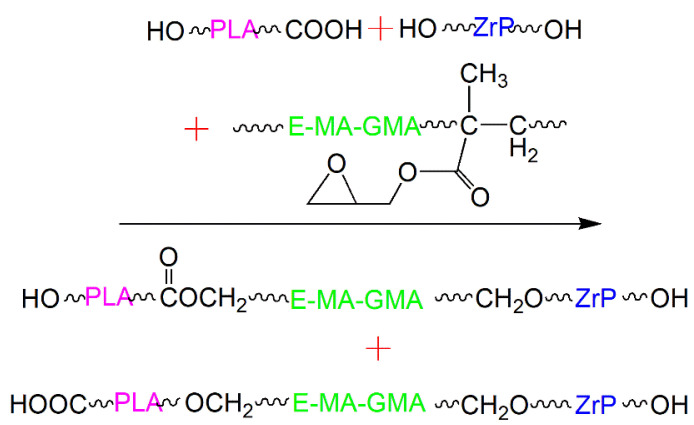
The reaction principle during melt-mixing between PLA, ZrP, and E-MA-GMA.

There are terminal carboxyl and hydroxyl groups at the ends of PLA molecular chains, hydroxy groups all around ZrP, and epoxy groups on the branched chains of E-MA-GMA. Therefore, in the process of melt-mixing in the mixing chamber, the terminal −OH and −COOH groups located on the PLA molecular chains will react with E-MA-GMA via the epoxy groups, while −OH groups around the periphery of ZrP nanofiller will react with the other epoxy groups of E-MA-GMA as well; the reaction mechanisms between PLA, E-MA-GMA, and ZrP are illustrated in Figure 1. In this way, the grafting between PLA and ZrP can be completed by elastomer E-MA-GMA through in situ reaction, which will promote the compatibility between PLA and ZrP, and enhance the interface combination between the two phases.

### 2.4. Characterization

#### 2.4.1. Scanning Electron Microscope

The microstructure of fractured sections of PLA/ZrP and PLA/ZrP/E-MA-GMA nanocomposites were studied by SEM (FEI-SEM, Hillsboro, OR, USA). The specimens were characterized under 5 kV accelerating voltage. To avoid electrostatic charging, a 0.1 nm thickness gold film was coated in the fractured surface of the specimen before SEM imaging.

#### 2.4.2. Transmission Electron Microscope

A TEM (JEOL 1200EX) was used to observe the dispersion morphology of the ZrP nanofillers and E-MA-GMA in the PLA matrix. The acceleration voltage used for the TEM test was 100 kV. All the specimens were stained by ruthenium tetroxide vapor and cryo-microtomed under −60 °C before TEM imaging.

#### 2.4.3. Fourier Transformation Infrared Spectroscopy

The FTIR absorption spectra of all the samples were recorded by a Perkin–Elmer Spectrum 2000 instrument, and the testing range was 400~4000 cm^−1^. The nanofillers, ZrP, were dried and ground together with KBr powder at room temperature. Then, the mixed powders were compressed into a disk for testing. The other samples were all compressed into slices at a temperature of 200 °C with a thickness of 120 μm employed for FTIR testing.

#### 2.4.4. Dynamic Mechanical Analysis

The prepared nanocomposites were cut into rectangular strips with dimensions of 10 mm × 4 mm × 40 mm to conduct dynamic mechanical analysis under the mode of three-point bending with 1 Hz scanning frequency via NETZSCH DMA 204C analyzer under strain amplitude of 0.05%. The temperature range and heating rate were −100~120 °C and 3 °C/min, respectively. The test was performed under a nitrogen atmosphere.

#### 2.4.5. Differential Scanning Calorimetry

A type of Netzsch 204F1 Phoenix DSC instrument was employed to analyze the thermal behavior of the prepared nanocomposites in the temperature range of 30~200 °C with 10 °C/min heating rate. The mass used for testing was about 6 mg. The formula for the crystallinity (*χ*_c_) calculation is shown as follows:

(1) $$\chi_{c}=\frac{\Delta H_{m}-\Delta H_{cc}}{w_{f}\Delta H_{m}^{0}}\times 100\%$$

where $\Delta H_{cc}$, $\Delta H_{m}$, and $\Delta H_{m}^{0}$ are the cold crystallization enthalpy, melting enthalpy, and theoretical melting enthalpy of 100% crystalline PLA ($\Delta H_{m}^{0}=$93.7 J/g) [31], and $w_{f}$ presents weight fraction of component PLA in composites.

#### 2.4.6. Thermogravimetric Analysis

The thermogravimetric analysis of PLA, E-MA-GMA, and the prepared PLA/ZrP and PLA/ZrP/E-MA-GMA nanocomposites was performed with a thermal analyzer (Netzsch STA-409c). An about 5 mg sample was tested in TGA equipment in a range of 30~600 °C. The heating speed was 20 °C/min and air flow was 50 cm^3^/min.

#### 2.4.7. Mechanical Properties

The prepared nanocomposites were cut into dumbbell shapes according to ASTM D882. The length, width, and thickness of the samples were 35 mm, 4 mm, and 1 mm, respectively. The tensile strengths of all the specimens were characterized by a universal testing machine (Instron 5566-type). The crosshead speed of the universal testing machine during testing process is 50 mm/min. Impact strength of all specimens were tested by a Zwick 5117 impact tester, according to the standard of GB/T 1843 (2008). The mechanical properties were tested at room temperature. All mechanical test results above were obtained by taking the average value of 5 test results.

## 3. Results and Discussion

### 3.1. Micro Morphology Analysis

**Figure 2:**
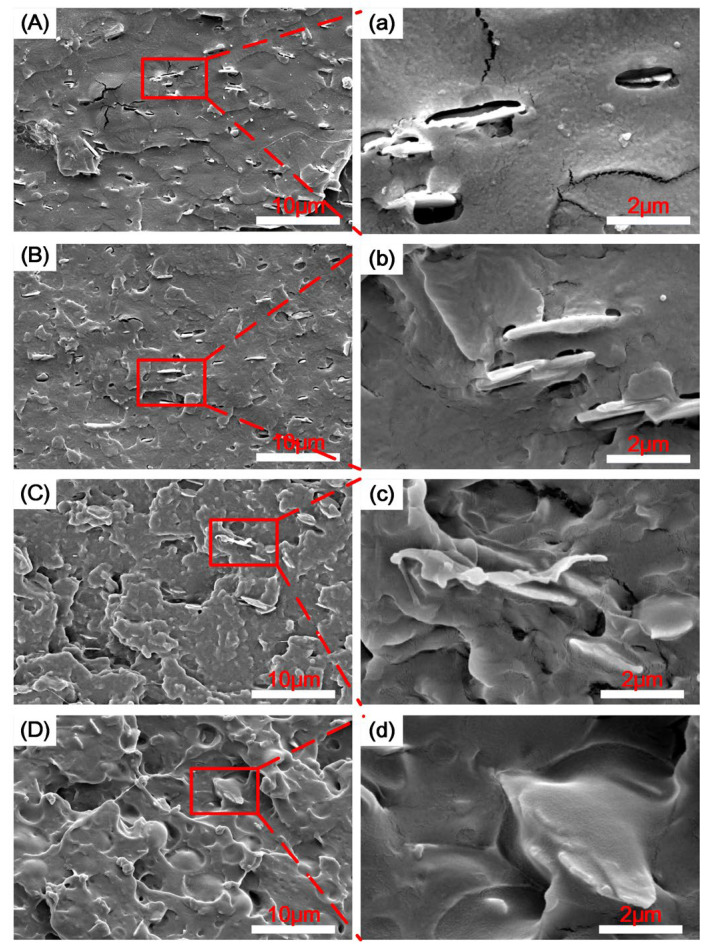
SEM micrographs of impact sections of PLA/ZrP/E-MA-GMA (wt/wt/wt) nanocomposites: (**A**) 97/3/0, (**B**) 92/3/5, (**C**) 82/3/15, and (**D**) 72/3/25. (**a**–**d**) are enlarged micrographs of (**A**–**D**), respectively.

The mechanical properties of PLA/ZrP and PLA/ZrP/E-MA-GMA nanocomposites are directly determined by their microstructures. So, it is necessary to use SEM and TEM to observe the impact sections of the prepared PLA/ZrP and PLA/ZrP/E-MA-GMA nanocomposites, which can reveal the micro dispersion morphology of the ZrP nanofillers and the elastomer E-MA-GMA in the PLA matrix, and thus characterize the toughening mechanism of the PLA/ZrP/E-MA-GMA nanocomposite via E-MA-GMA. Figure 2 shows SEM micrographs of the impact sections of the PLA/ZrP and PLA/ZrP/E-MA-GMA nanocomposites.

As it is known, pure PLA is a typical brittle material, which is not resistant to external impacts and its impact section is both flat and smooth [32]. From Figure 2A,a, we can see that the impact section of the PLA/ZrP nanocomposite is smooth and neat, similar to pure PLA. It is obvious that there are two phase separation interfaces between the lamella of the ZrP nanofillers and the matrix in Figure 2a, which verified that the lamella of the ZrP nanofiller is incompatible with PLA and its addition has no effect on improving the toughening of PLA. As a result, the PLA/ZrP nanocomposite still shows brittle fracturing. Figure 2B,b are SEM micrographs of the PLA/ZrP/E-MA-GMA nanocomposite with 5% content of E-MA-GMA. From the two SEM pictures we can see that a small amount of E-MA-GMA can ameliorate the compatibility between ZrP nanofillers and the matrix of PLA, but there are still some interfaces between the two phases. Hence, the impact section of the PLA/ZrP/E-MA-GMA nanocomposite with 5% E-MA-GMA still shows brittle fracturing. When the E-MA-GMA content is 15%, the ZrP nanofillers were all wrapped by E-MA-GMA in PLA matrix, and there are no interfaces that can be seen between PLA and ZrP, as is shown in Figure 2C,c. So, the impact-fracture form of the nanocomposite changed from brittle to ductile, which enables the absorption of the external impact force and greatly improves its impact resistance. We can see from Figure 2D,d that there are more E-MA-GMA wrapped around the nanofillers and that part of the E-MA-GMA dispersed in the matrix PLA with the further increase in the E-MA-GMA content. Further plastic deformations occured in the impact sections and the toughness of the PLA/ZrP/E-MA-GMA nanocomposite was further improved.

**Figure 3:**
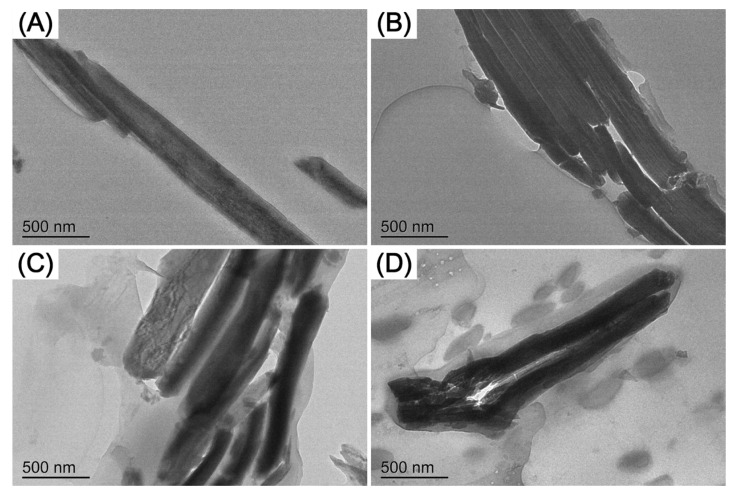
TEM micrographs of PLA/ZrP/E-MA-GMA (wt/wt/wt) blends with different compositions: (**A**) 97/3/0, (**B**) 92/3/5, (**C**) 82/3/15, and (**D**) 72/3/25.

The microstructural morphology of the E-MA-GMA and ZrP nanofillers dispersed in the PLA matrix was further characterized by TEM in Figure 3. Through the comparison of Figure 3A with Figure 3B–D, we can see that the ZrP nanofillers were wrapped by the elastomer E-MA-GMA when dispersing in the PLA matrix, forming a typical core–shell morphology, which cannot be seen in Figure 3A. With the increase in the E-MA-GMA content, the ZrP nanofillers are wrapped by an increasing amount of E-MA-GMA, which corresponds to the SEM micrographs and further verifies the results of the analysis above.

### 3.2. Fourier Transformation Infrared Spectroscopy

**Figure 4:**
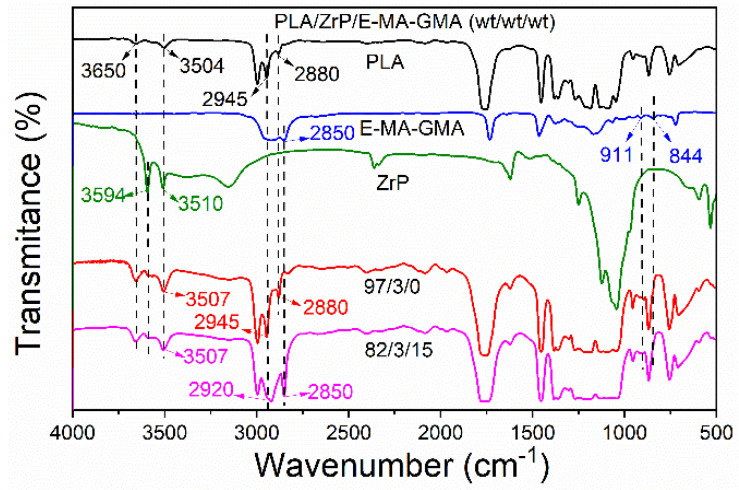
FTIR curves of PLA, E-MA-GMA, ZrP, PLA/ZrP and PLA/ZrP/E-MA-GMA.

Figure 4 shows the FTIR spectral curves of the pure PLA, E-MA-GMA, ZrP, PLA/ZrP, and PLA/ZrP/E-MA-GMA nanocomposites in the range of 500~4000 cm^−1^. An analysis of the curves of the FTIR was performed to prove the compatible reactions between PLA, E-MA-GMA, and ZrP. It is known that PLA and ZrP both contain hydroxyl groups: the characteristic hydroxyl peaks of PLA are at 3650 cm^−1^ and 3504 cm^−1^, while the hydroxyl peaks for the ZrP nanofiller are located at 3510 cm^−1^ and 3594 cm^−1^. Methyl and epoxy groups are characteristic groups of E-MA-GMA: the characteristic peak of its methyl group appears at 2850 cm^−1^ and its epoxy group peaks are around 844 cm^−1^ and 911 cm^−1^.

By comparing the FTIR curves of pure PLA, ZrP, and PLA/ZrP nanocomposites, we can see that there are no characteristic peaks disappearing in the FTIR spectral curve of the PLA/ZrP nanocomposite, which indicates that the PLA matrix cannot react with ZrP nanofiller. It can be seen from Figure 4 that there are no characteristic peaks of the epoxy group at 844 cm^−1^ and 911 cm^−1^ in the FTIR spectral curve of the PLA/ZrP/E-MA-GMA nanocomposite. The symmetrical and asymmetrical stretching vibration absorption peaks of the methyl group are located at 2850 cm^−1^ and 2920 cm^−1^, which can be found in the FTIR curve of the PLA/ZrP/E-MA-GMA nanocomposite. The results above indicate that the −OH and −COOH groups of PLA reacted with E-MA-GMA through its epoxy groups. This chemical reaction has also been confirmed by Wu [33]. By comparing the FTIR spectral curve of the PLA/ZrP with the PLA/ZrP/E-MA-GMA, it is evident that the intensity of the stretching vibration peak of the −OH group at 3507 cm^−1^ in the PLA/ZrP/E-MA-GMA nanocomposite is lower than that of the PLA/ZrP nanocomposite, which indicates that when E-MA-GMA mixes into the PLA/ZrP nanocomposite there also reactions between the −OH groups around ZrP and the epoxy groups in the E-MA-GMA molecular chains.

### 3.3. Dynamic Mechanical Analysis

**Figure 5:**
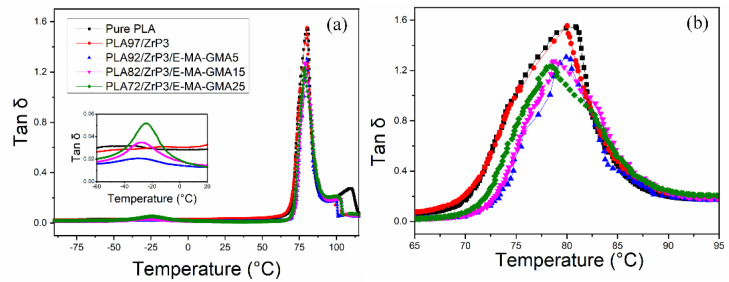
Tan δ curves of pure PLA, PLA/ZrP, and PLA/ZrP/E-MA-GMA nanocomposites in the ranges of (**a**) −90 °C to 115 °C and (**b**) 65 °C to 95 °C.

DMA is an effective method for assessing the miscibility between E-MA-GMA and the PLA matrix. The miscibility of the two polymers determines the microstructure of the PLA/ZrP/E-MA-GMA nanocomposite, which directly influences its mechanical properties. The glass transition temperatures (T_g_) of pure PLA, PLA/ZrP, and PLA/ZrP/E-MA-GMA are listed in Table 1 and the Tan δ curves are shown in Figure 5.

As shown in Figure 5, there is only one peak for the pure PLA and PLA/ZrP nanocomposite, while the PLA/ZrP/E-MA-GMA nanocomposites all have two peaks. One of the peak values in the high temperature range is the glass transition temperature (T_g2_) of PLA, and the other one at the low temperature range is the glass transition temperature (T_g1_) of E-MA-GMA. ZrP is an inorganic filler, so it has no glass transition temperature, and the PLA/ZrP nanocomposite has only one peak that corresponds to the T_g_ of the PLA matrix. For the PLA/ZrP/E-MA-GMA nanocomposites, with the increase in the E-MA-GMA content, the T_g1_ in the low temperature zone increases gradually, while the T_g2_ in the high temperature zone decreases gradually. This phenomenon illustrates that the elastomer E-MA-GMA and PLA have good compatibility, and their compatibility increases with the increasing content of E-MA-GMA. This result confirms the previously speculated reaction compatibilization, which is consistent with the conclusions of Zhang [34,35], Li [36], and Wei [37].

### 3.4. Melting and Crystalline Behavior

**Figure 6:**
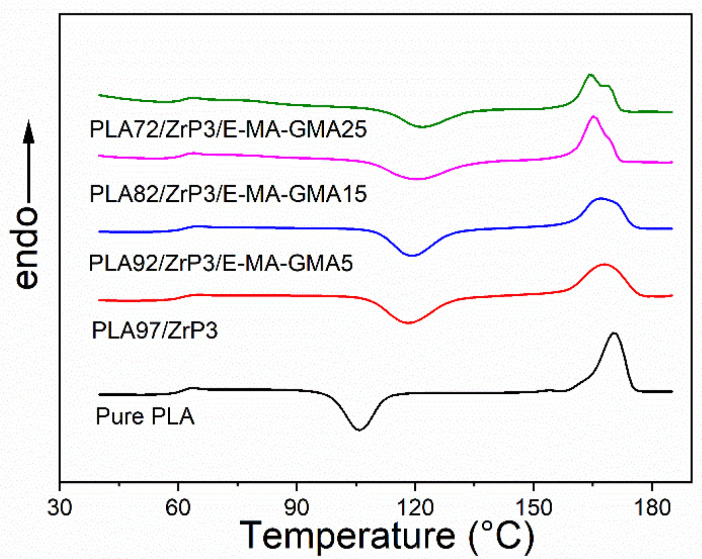
DSC curves of melting for pure PLA, PLA/ZrP, and PLA/ZrP/E-MA-GMA nanocomposites.

Figure 6 shows the melting behaviors of the PLA, PLA/ZrP, and PLA/ZrP/E-MA-GMA nanocomposites characterized by DSC. The detailed data of the DSC results are listed in Table 2, including the T_g_, T_cc_ (cold crystallization temperature), T_m_ (melting temperature), ΔH_cc_, ΔH_m_, and χ_c_.

From Table 2 and Figure 6 we can see that the ZrP nanofiller and E-MA-GMA have almost no effect on the T_g_ of the PLA matrix, as the value of T_g_ of the PLA/ZrP and PLA/ZrP/E-MA-GMA nanocomposites changed only slightly compared with pure PLA. The results obtained by Equation (1) show that ZrP can reduce the crystallinity of the PLA; consequently, the T_cc_ was improved by 12.4 °C, from 105.8 °C to 118.2 °C of the PLA/ZrP nanocomposite, and the melting temperature decreased slightly. This is because the lamella of the ZrP nanofiller can restrain the crystallization of the PLA’s molecular chains. As for the PLA/ZrP/E-MA-GMA nanocomposites, the degree of crystallinity was further decreased after the addition of E-MA-GMA. With the increase in the content of E-MA-GMA, the degree of the crystallinity of the nanocomposite decreased continuously, and when the content was 25%, the crystallinity was only 2.8%, far below that of pure PLA. Moreover, double melting endotherms can be seen for the PLA/ZrP/E-MA-GMA samples. The reason for this phenomenon is the destruction of the PLA molecular chain’s regularity by E-MA-GMA and ZrP. During the heating process, the part of the PLA with imperfect crystals was melted first; then, the PLA with more perfect crystals was remelted, thereby leading to the appearance of double peaks. In addition, the cold crystallization temperature of the PLA/ZrP and PLA/ZrP/E-MA-GMA nanocomposites increases with the increased content of E-MA-GMA, while the melting temperature decreases accordingly. This trend can be explained by the fact that the mobility of the PLA’s molecular chains are limited by the strong interfacial interaction formed by compatible reactions between the ZrP nanofillers, E-MA-GMA, and the PLA matrix. Therefore, mixing a certain amount of E-MA-GMA into the PLA/ZrP nanocomposite can not only reduce the crystallinity but also increase the cold crystallization temperature.

### 3.5. Thermogravimetric Analysis

**Figure 7:**
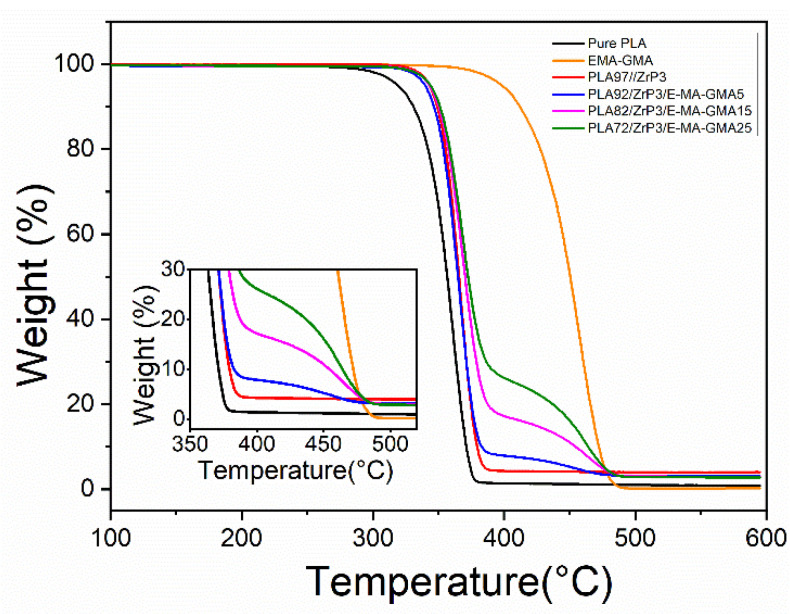
TGA curves of PLA, E-MA-GMA, PLA/ZrP, and PLA/ZrP/E-MA-GMA nanocomposites.

Figure 7 shows the thermogravimetric curves for the pure PLA, PLA/ZrP, and PLA/ZrP/E-MA-GMA nanocomposites, which demonstrate the influence of the ZrP nanofillers and E-MA-GMA on the thermogravimetry of the PLA and PLA/ZrP nanocomposites, respectively. Table 3 presents the initial decomposed temperature (*T*_i_), maximum rate of weight loss temperature (*T*_p_), final decomposed temperature (*T*_f_), maximum rate of mass loss (*R*_max_), and fraction of the residue remaining at 590 °C (Char) of the pure PLA, E-MA-GMA, PLA/ZrP, and PLA/ZrP/E-MA-GMA nanocomposites.

It can be seen from Figure 7 that the lamellar ZrP nanofillers improved the thermal stability of PLA significantly. The onset of thermal degradation and the final decomposed temperature of pure PLA were 319.0 °C and 373.6 °C, and the content of carbon residue was 0.82%. When adding 3% content of ZrP into PLA, the initial and final decomposed temperatures were 342.9 °C and 378.4 °C, the Char% was 4.04%, improved by 23.9 °C, 4.8 °C, and 3.22%, respectively, compared with that of PLA. The results verified that ZrP nanofillers can improve the thermal stability of PLA through the mixing method.

From Table 3 and Figure 7, we can also see that the E-MA-GMA exhibits an excellent thermal stability: the initial and final decomposed temperatures are 398.4 °C and 491.5 °C, respectively, both above that of PLA. By adding E-MA-GMA into the PLA/ZrP nanocomposite, the thermal stability can be further improved. When the additional E-MA-GMA content was 15%, the R_max_ of the PLA/ZrP/E-MA-GMA nanocomposite reduced from 35.98%/min to 25.43% compared with the PLA/ZrP nanocomposite, which effectively slows down the thermal degradation rate.

### 3.6. Mechanical Properties

**Figure 8:**
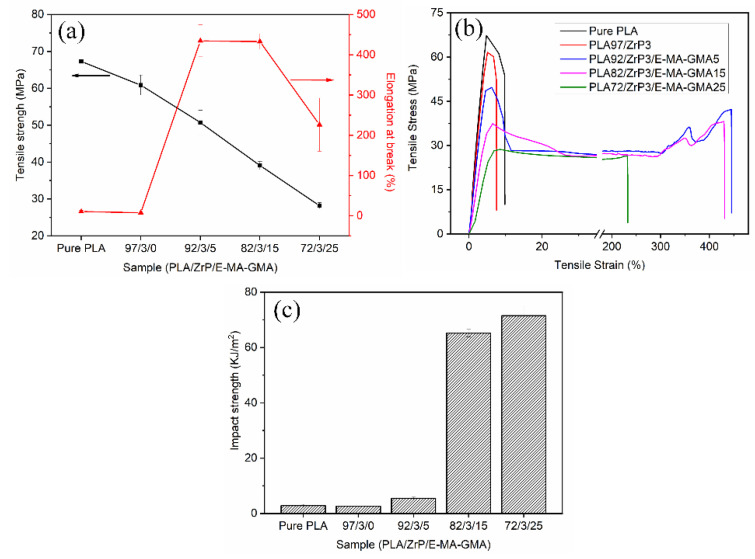
Mechanical properties of PLA, PLA/ZrP, and PLA/ZrP/E-MA-GMA nanocomposites: (**a**) tensile strength and elongation at break, (**b**) stress–strain curves, and (**c**) impact strength.

Figure 8 presents the mechanical properties of pure PLA and the prepared PLA/ZrP and PLA/ZrP/E-MA-GMA nanocomposites. Figure 8a exhibits the change curve of the elongation at break and tensile strength with different components, Figure 8b exhibits the stress–strain curves, and Figure 8c exhibits the impact strength of pure PLA and the prepared nanocomposites, respectively. The results of the mechanical properties are listed in Table 4. 

From Figure 8, we can see that mixing 3% content of ZrP nanofiller into the PLA matrix has few effect on changing the brittleness and improving the toughness of PLA. The physical and mechanical properties of the PLA/ZrP nanocomposite are all worse than pure PLA. This is mainly because the ZrP nanofiller is incompatible with the PLA matrix, and the interface combination between the two is weak. When the specimen is impacted by external force, the ZrP nanofiller induces a stress concentration point, which can easily destroy the nanocomposite. Thus, the mechanical properties of PLA will be reduced. Therefore, it is not suitable to mix ZrP nanofiller into the PLA matrix alone.

From Figure 8b,c, we can see that the elastomer E-MA-GMA can effectively ameliorate the toughness of the PLA/ZrP nanocomposite, which allows the PLA/ZrP nanocomposite to transform from a brittle to tough fracture type. When the E-MA-GMA content is 15%, the elongation at break of the PLA/ZrP/E-MA-GMA nanocomposite rises sharply to 432.8%, almost 60 times higher than that of the PLA/ZrP nanocomposite. The PLA/ZrP/E-MA-GMA nanocomposite also has excellent impact resistance, which can be seen from Figure 8c. The impact strength of the PLA/ZrP/E-MA-GMA nanocomposite is 5.5 kJ/m^2^ when the mixing content of E-MA-GMA is 5%, two times higher than that of the PLA/ZrP nanocomposite. When the E-MA-GMA content increases to 15%, the impact strength of the PLA/ZrP/E-MA-GMA nanocomposite is sharply improved to 65.2 kJ/m^2^. With the continuous increase in the E-MA-GMA content to 25%, its impact strength is further increased to 71.5 kJ/m^2^. Whereas the tensile strength and modulus decreases with the increase in the content of E-MA-GMA. Although the content of E-MA-GMA is 25%, the PLA/ZrP/E-MA-GMA nanocomposite still has a high tensile strength of 28.2 MPa.

The reason for the PLA/ZrP nanocomposite’s transference from a brittle to ductile fracture type via the addition of E-MA-GMA, and why the prepared PLA/ZrP/E-MA-GMA nanocomposite has a high impact strength, is that the epoxy group of E-MA-GMA can react with the terminal carboxyl and hydroxyl groups of PLA and the hydroxyl group of ZrP during the mixing process, which improves the combination between the PLA and ZrP. In addition, E-MA-GMA is an elastomer; thus, it can synergize with ZrP in the PLA matrix to absorb the external impact force and enhance the impact strength.

## 4. Conclusions

In this paper, super-tough PLA/ZrP/E-MA-GMA nanocomposites were fabricated via the reactive melt-mixing method. With the addition of 15% E-MA-GMA content, the nanocomposite showed the best comprehensive performance: the initial decomposed temperature improved by 319 °C to 342.9 °C; the impact strength and elongation at break improved 22.5 and 41.6 times that of pure PLA, respectively; and the tensile strength still maintained 39.1 MPa, all of which show excellent comprehensive mechanical properties. The FTIR spectra verified that E-MA-GMA can react with PLA and ZrP during mixing process, which increases the compatibility between PLA and ZrP. The SEM and TEM images revealed the micromorphology, whereby the ZrP nanofillers were well-wrapped by the elastomer E-MA-GMA in the PLA matrix and formed a typical core–shell structure, which granted the prepared nanocomposite excellent toughness. 

## Figures and Tables

**Table 1:** T_g_ of pure PLA, PLA/ ZrP and PLA/ZrP/E-MA-GMA nanocomposites tested by DMA.

Compositions	T_g1_ (°C)	T_g2_ (°C)
pure PLA	—	80.4
PLA/ZrP (97/3)	—	80.0
PLA/ZrP/E-MA-GMA (92/3/5)	−29.9	79.9
PLA/ZrP/E-MA-GMA (82/3/15)	−27.5	78.7
PLA/ZrP/E-MA-GMA (72/3/25)	−24.2	78.3

**Table 2:** The *T_g_, T_cc_, T_m_, *Δ*H_cc_, *Δ*H_m_*, and *χ_c_* of PLA, PLA/ZrP and PLA/ZrP/E-MA-GMA nanocomposites.

Compositions	*T*_g_ (°C)	*T_cc_* (°C)	*T*_m_ (°C)	Δ*H*_cc_ (J/g)	Δ*H*_m_ (J/g)	*χ*_c_ (%)
pure PLA	60.3	105.8	170.3	32.4	40.6	8.8
PLA/ZrP (97/3)	60.2	118.2	168.1	30.0	34.9	5.4
PLA/ZrP/E-MA-GMA (92/3/5)	60.9	119.0	167.0	26.0	30	4.6
PLA/ZrP/E-MA-GMA (82/3/15)	60.9	120.5	165.3	28.1	31.1	3.9
PLA/ZrP/E-MA-GMA (72/3/25)	61.0	121.6	164.5	23.3	25.2	2.8

**Table 3:** TGA results of PLA, E-MA-GMA, PLA/ZrP, and PLA/ZrP/E-MA-GMA nanocomposites.

Compositions	*T*_i_ (°C)	*T*_p_ (°C)	*T*_f_ (°C)	*R*_max_ (%/min)	Char (%)
pure PLA	319.0	362.4	373.6	29.63	0.82
E-MA-GMA	398.4	457.5	491.5	20.37	0.23
PLA/ZrP (97/3)	342.9	368.2	378.4	35.98	4.04
PLA/ZrP/E-MA-GMA (92/3/5)	339.8	367.2	472.1	32.15	3.07
PLA/ZrP/E-MA-GMA (82/3/15)	342.9	370.5	484.9	25.43	2.96
PLA/ZrP/E-MA-GMA (72/3/25)	343.8	369.9	472.3	23.31	2.77

**Table 4:** The detailed data of mechanical properties for PLA, PLA/ZrP, and PLA/ZrP/E-MA-GMA nanocomposites.

Compositions	Tensile Strength (MPa)	Tensile Modulus (MPa)	Impact Strength (kJ/m^2^)	Elongation at Break (%)
pure PLA	67.3 ± 0.1	1590 ± 21	2.9 ± 0.4	10.4 ± 2.8
PLA/ZrP (97/3)	60.9 ± 2.7	1218 ± 45	2.6 ± 0.1	7.4 ± 0.7
PLA/ZrP/E-MA-GMA (92/3/5)	50.7 ± 3.3	1177 ± 51	5.5 ± 0.4	434.7 ± 39.8
PLA/ZrP/E-MA-GMA (82/3/15)	39.1 ± 1.1	824 ± 40	65.2 ± 1.4	432.8 ± 19.1
PLA/ZrP/E-MA-GMA (72/3/25)	28.2 ± 0.8	604 ± 48	71.5 ± 3.7	226.3 ± 66.3

## Data Availability

The data presented in this study are available from the listed authors.
